# Shylock's Argument out of Court

**Published:** 1918-06-15

**Authors:** 


### "A NURSE'S CHARTER OF LIBERTY."

### Shylock's Argument Out of Court.

I regret to have to express disappointment with Lord Knutsford's reply to my criticism of his Nurse's Charter of Liberty. I asked him a question, and he responds with an evasion of the issue. The question was—Why, above all other workers, nurses should be asked to forfeit their freedom? His reply is that the language used is "ridiculous," and that if there is an agreement there is no forfeiture. May I say that this appears to me a petulant answer, and unworthy of a champion of the profession? "Nurses, like every other person," remarks Lord Knutsford, "are free to enter the service they select, and free to leave it if they do not like it." In other words, an agreement is a justification. Now, with great respect, an agreement is not a justification. An agreement was the argument of Shylock. "My bond!" An agreement was the argument of the slave-owners. The slaves preferred their slavery. Parliament has made many enactments to render such agreements impossible, including the Factories Acts, and Acts relating to the employment of children. On the same principle the sale of drink is restricted, and vice is driven from the streets. The law has vetoed the right of the citizen to barter his body and his soul, and however far Lord Knutsford may dissociate himself from the classes against whom these preventive laws have been made, his defence of the long hours and scanty freedom of the nurse is identical with that of Shylock ancient and modern. In these two words is concentrated the essence of their code—"My bond!"

I think more highly of Lord Knutsford than to believe he will stand by that argument, which I have no doubt he offered in haste. My point is that the world of British workers demand and enjoy one day's rest in seven plus a half-day's rest in seven, and I asked him to state exactly why this freedom should be denied to the British hospital nurse. My reason for appealing to him in particular was twofold. He is chairman to the largest hospital in the kingdom, a hospital which regards itself, and not without justification, as setting an example to the others. Moreover, he had already considered the whole question with such obvious care as to prepare a Charter of Liberty—a standard for the guidance of probationers. In these circumstances I venture to submit that Lord Knutsford owes it to British nurses to state why he expects them, above all other workers, to be content with conditions which by comparison are conspicuously harsh. The chairman of the "London" knows well enough that the remedy lies in the employing of a larger staff. If, for example, 140 nurses are required where one day's rest in seven is denied, it is clear that if 160 are employed such a privilege might be granted, and if 170 are employed the weekly half-day off could also be granted. That the maximum rather than the minimum should be granted is demonstrated by Lord Knutsford's own words. The nurse's life is hard, he admits, because of "the constant keeping the mind 'at attention.'" Is it not perfectly plain that relaxation is not only the best but the only cure? The *joie de vivre* for the worker depends altogether on reasonable rest and recreation. For the woman worker, and especially the hospital woman worker, the supreme consideration is to be able to get away from it all for a spell, to have it out of sight and out of mind, to go where she wills and do as she pleases. Lord Knutsford's Charter fails to provide such freedom, and the more liberal terms of the London Hospital are little better.

I am particularly anxious to have my question frankly answered. My criticism is not captious, nor do I seek to misrepresent the best of employers. Moreover, I am not out for a controversial victory. But I want to establish in the minds of others that which is fully established in my own—that the hospital nurse toils longer and at a lower wage than her fellows similarly equipped—that she sacrifices her pleasure, her time, and her talent in the public service, and that, therefore, the public owe her a substantial debt. I do not seek to condemn Lord Knutsford. I have already said this; I repeat it, and I mean it. On the contrary, I want to convince him. If he is convinced, he is big enough and generous enough to champion the cause of the nurse, and to help to impress on the British people that the facts are as stated. What Lord Knutsford says to-day other hospital chairmen and hospital boards will echo to-morrow. The hospital nurse is entitled to a career, to the prizes of a career, to the joyousness of a happy life, to security of tenure within reasonable limits, and to freedom from anxiety when her life's work is done. I decline to believe that the British public will deny her all this. When the truth is brought home to them they will write her a new Charter in letters of gold.

IERNE.

